# Zinc finger protein 91 (ZFP91) activates HIF-1α *via* NF-κB/p65 to promote proliferation and tumorigenesis of colon cancer

**DOI:** 10.18632/oncotarget.9070

**Published:** 2016-04-28

**Authors:** Juan Ma, Chunliu Mi, Ke Si Wang, Jung Joon Lee, Xuejun Jin

**Affiliations:** ^1^ Key Laboratory of Natural Resources of Changbai Mountain & Functional Molecules, Ministry of Education, Molecular Medicine Research Center, College of Pharmacy, Yanbian University, Yanji 133002, Jilin Province, China

**Keywords:** ZFP91, HIF-1α, NF-κ B/p65, tumorigenesis, colon cancer

## Abstract

Zinc finger protein 91 (ZFP91) has been reported to be involved in various biological processes. However, the clinical significance and biological role of ZFP91 in colon cancer remains unknown. Here, we show that ZFP91 expression is upregulated in patients with colon cancer. We found that ZFP91 upregulated HIF-1α at the levels of promoter and protein in colon cancer cells. Using chromatin immunoprecipitation, electrophoretic mobility shift assay and luciferase reporter gene assay, we found that NF-κB/p65 is required for the binding of ZFP91 to the HIF-1α promoter at −197/−188 base pairs and for the transcriptional activation of HIF-1α gene mediated by ZFP91. Flow cytometry, 5-ethynyl-2′-deoxyuridine (EdU) incorporation and tumor xenograft assay demonstrated that ZFP91 enhanced cell proliferation of colon cancer through upregulating HIF-1α *in vitro* and *in vivo*. Furthermore, ZFP91 is positively associated with HIF-1α in human colon cancer. Thus, we concluded that ZFP91 activates transcriptional coregulatory protein HIF-1α through transcription factor NF-κB/p65 in the promotion of proliferation and tumorigenesis in colon cancer cell. ZFP91 may serve as a driver gene to activate HIF-1α transcription in the development of cancer.

## INTRODUCTION

Zinc finger protein (ZFP91), a conserved 63.5kDa nuclear protein with structural motifs characteristic of transcription factor, contain five zinc-finer domains, one leucine-zipper pattern, one coiled-coil structure, and several nuclear localization signals [1]. It has been reported that ZFP91 interacts with ARF tumor suppressor (cyclin-dependent kinase inhibitor 2A), which is known for its induction of p53-dependent cell death or growth arrest in response to activated oncogenes [2]. Furthermore, ZFP91 plays a critical role in acute myelogenous leukemia and prostate pathology [3, 4]. Our previous study indicated that ZFP91 is an atypical E3 ligase activating NF-κB-inducing kinase (NIK) *via* Lys^63^-linked ubiquitination in the noncanonical NF-κB signaling pathway [5, 6]. However, the underlying mechanism of ZFP91 in tumorigenesis has not been well defined.

Tumor hypoxia activates a battery of genes that lead to angiogenesis, metastasis, drug resistance and tumor invasion by stabilizing HIF-1 [7]. HIF-1 is a heterodimeric transcription factor composed of α and β subunits [8]. Although HIF-1β is constitutively expressed, HIF-1α is tightly controlled by oxygen level. HIF-1α is a central molecule involved in mediating these effects of hypoxia. In colorectal cancer, hypoxia stabilizes the transcription factor HIF-1α, leading to the expression of genes that are involved in tumor vascularization, metastasis/migration, cell survival and chemo-resistance [9, 10]. Therefore, HIF-1α is a rational target for the development of new therapeutics for colorectal cancer.

Under normoxia, the HIF-1α gene is continuously transcribed and translated, but its level is very low due to rapid degradation *via* the ubiquitin-proteasomal pathway mediated by prolyl hydroxylase [11]. In contrast, hypoxia inhibits prolyl hydroxylase activity and consequently results in the accumulation of HIF-1α protein [12]. Accumulated HIF-1α translocates to nuclei and dimerizes with HIF-1β to form a functional transcription factor capable of DNA binding at hypoxia response elements (HREs) and the transcriptional activation of target genes [13, 14]. Although the oxygen-dependent regulation of degradation is the primary mechanism of HIF-1α accumulation, HIF-1α can also be regulated at the levels of transcription and translation [13, 15]. Many of the stimuli that induce HIF-1α in normoxia and even short-term hypoxia are known to activate a number of other transcription factors such as NF-κB. It is therefore plausible that cross-talk between these two transcription factors can occur. In fact, phosphorylation of IκB and subsequent activation of the NF-κB subunits p65 has been reported to contribute to be basal levels of HIF-1α mRNA and protein, and to mediate HIF-1α expression and promoter activity in response to thrombin, H_2_O_2_ and even short-term hypoxia [16–18]. The HIF-1α promoter contains NF-κB-binding sites, some of which have not been functionally well characterized [19–21]. In this study, we found that ZFP91 expression was significantly upregulated in colon cancer cells and tissues. Therefore, we asked how ZFP91 can promote proliferation and tumorigenesis in colon cancer cells *in vitro* and *in vivo*. Here we demonstrated that ZFP91 was mechanistically associated with the NF-κB/p65 in the expression of HIF-1α. Furthermore, ZFP91 is positively associated with HIF-1α in human colon cancer. Our results show that ZFP91 functions as an oncoprotein in the colon cancer progression and may provide a new prognostic marker and therapeutic target for the treatment of colon cancer.

## RESULTS

### ZFP91 is positively associated with HIF-1α and up-regulated in human colon cancer

**Figure 1:**
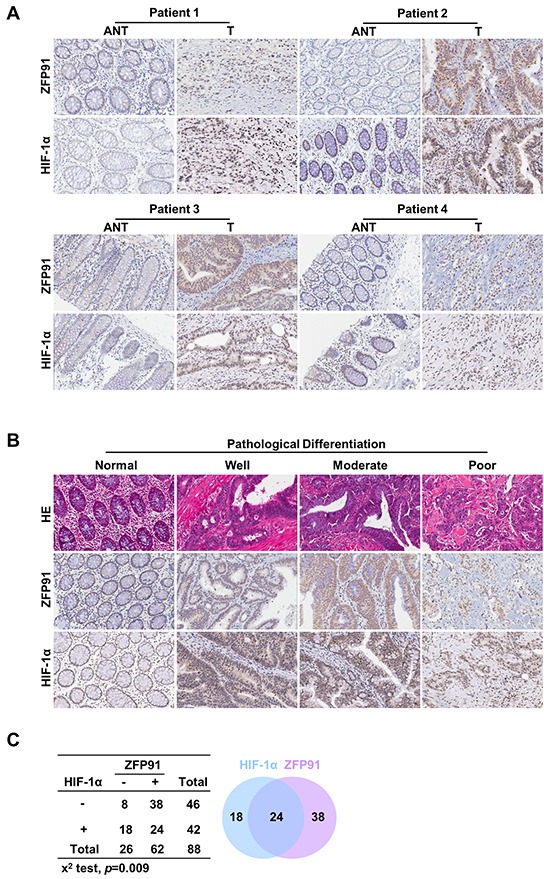
Up-regulation of ZFP91 and HIF-1α in colon cancer patients correlates with poor prognosis **A, B.** H&E and IHC stainings of ZFP91 and HIF-1α expression (A) in matched primary colon cancer tissues (T) and adjacent noncancerous tissues (ANT) and (B) in normal colon tissues and colon cancer tissues with different degrees of differentiation. Original magnification, 200x. **C.** Hyperexpression of ZFP91 and HIF-1α in overlapping human colon cancer (p=0.009, χ2test).

To investigate the relationship between ZFP91 expression and the clinical features of colon cancer, ZFP91 expression was examined in 88 paraffin-embedded, archived colon cancer tissues using IHC staining. The samples included 7 cases of clinical stage I(8.0%), 37 cases of clinical stage II(42.0%), 36 cases of clinical stage III(40.9%), and 8 cases of clinical stage IV(9.1%) colon cancer. The human colon cancer specimens exhibited a robust expression of ZFP91 and HIF-1α compared with adjacent tissues (Figure 1A). Statistical analyses of the IHC staining are summarized in Supplementary Tables S1 and S2. Furthermore, IHC staining showed that ZFP91 and HIF-1α expression in the tumors is closely correlated with a poor pathologic differentiation (Figure 1B). Moreover, in 27.27% of human colon cancer, ZFP91 and HIF-1α were collectively up-regulated (Figure 1C, *p*=0.009). These results support the mechanistic and clinical significance of the ZFP91-HIF-1α axis may be as an effective biomarker for human colon cancer diagnosis and prognosis evaluation.

### ZFP91 up-regulates HIF-1α in colon cancer cells

**Figure 2:**
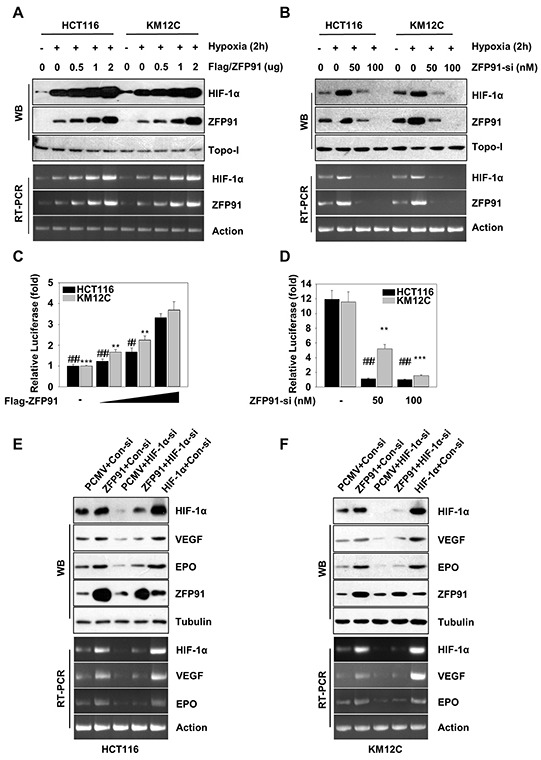
ZFP91 upregulates HIF-1α in colon cancer cells **A, B.** HCT116 and KM12C cells transfected with Flag-ZFP91 or ZFP91 siRNA were stimulated with hypoxia for final 2 h. The protein and mRNA expression levels of HIF-1α and ZFP91 were determined by western blot and RT-PCR, respectively. **C, D.** HCT116 and KM12C cells transfected with Flag-ZFP91 or ZFP91 siRNA together with HIF-1α promoter reporter construct pGL-WT-HIF-1α were stimulated with hypoxia for final 2 h, and then the cell lysates were subject to the measurement of dual luciferase activity. Data represented as mean ± standard deviation of three independent experiments. (^**^p<0.01, ^***^p<0.001,^##^*p*<0.01,^###^*p*<0.001). **E, F.** HCT116 and KM12C cells pre-treated with PCMV, Flag-ZFP91 or HA-HIF-1α together with control siRNA or HIF-1α siRNA stimulated with hypoxia for final 2 h. The cell lysates were analyzed using indicated antibodies. The mRNA expression levels of HIF-1α VEGF and EPO were determined by RT-PCR.

HIF-1α is highly expressed in a large number of human primary and metastatic tumor tissues [22]. To explore the mechanism by which ZFP91 accelerates cell proliferation and tumorigenesis, we tested whether ZFP91 affects HIF-1α signaling. Intriguingly, overexpression of ZFP91 increased the hypoxia-induced expression of HIF-1α protein and mRNA levels dose-dependently in HCT116 and KM12C cells (Figure 2A), whereas the knockdown of ZFP91 by siRNA decreased the hypoxia-induced expression of HIF-1α protein and mRNA levels (Figure 2B). To assess the effect of ZFP91 on HIF-1α promoter activity, we cloned the promoter region of HIF-1α (−625/+283) into PGL-3-basic plasmid according to the report [19]. Luciferase reporter gene assays showed that the overexpression or knockdown of ZFP91 could increase or decrease hypoxia-induced the promoter activity of HIF-1α dose-dependently (Figure 2C, 2D). The expression of VEGF and EPO involved in colon cancer cells proliferation, angiogenesis, invasion and metastasis [7, 13, 23]. We further examined the effect of ZFP91 on VEGF and EPO expressions. Western blot and RT-PCR analysis revealed that the overexpression of ZFP91 upregulated the expression of VEGF and EPO protein and mRNA levels in HCT116 and KM12C cells (Figure 2E and 2F, lane 2). Naturally, the overexpression of HIF-1α upregulated VEGF and EPO protein and mRNA levels in the cells (Figure 2E and 2F, lane 5), silencing of HIF-1α abolished the upregulation of VEGF and EPO protein and mRNA levels mediated by ZFP91 (Figure 2E and 2F, lane 4), supporting that ZFP91 is able to upregulate HIF-1α in colon cancer cells.

### ZFP91 activates HIF-1α promoter through interacting with NF-κB/p65

**Figure 3:**
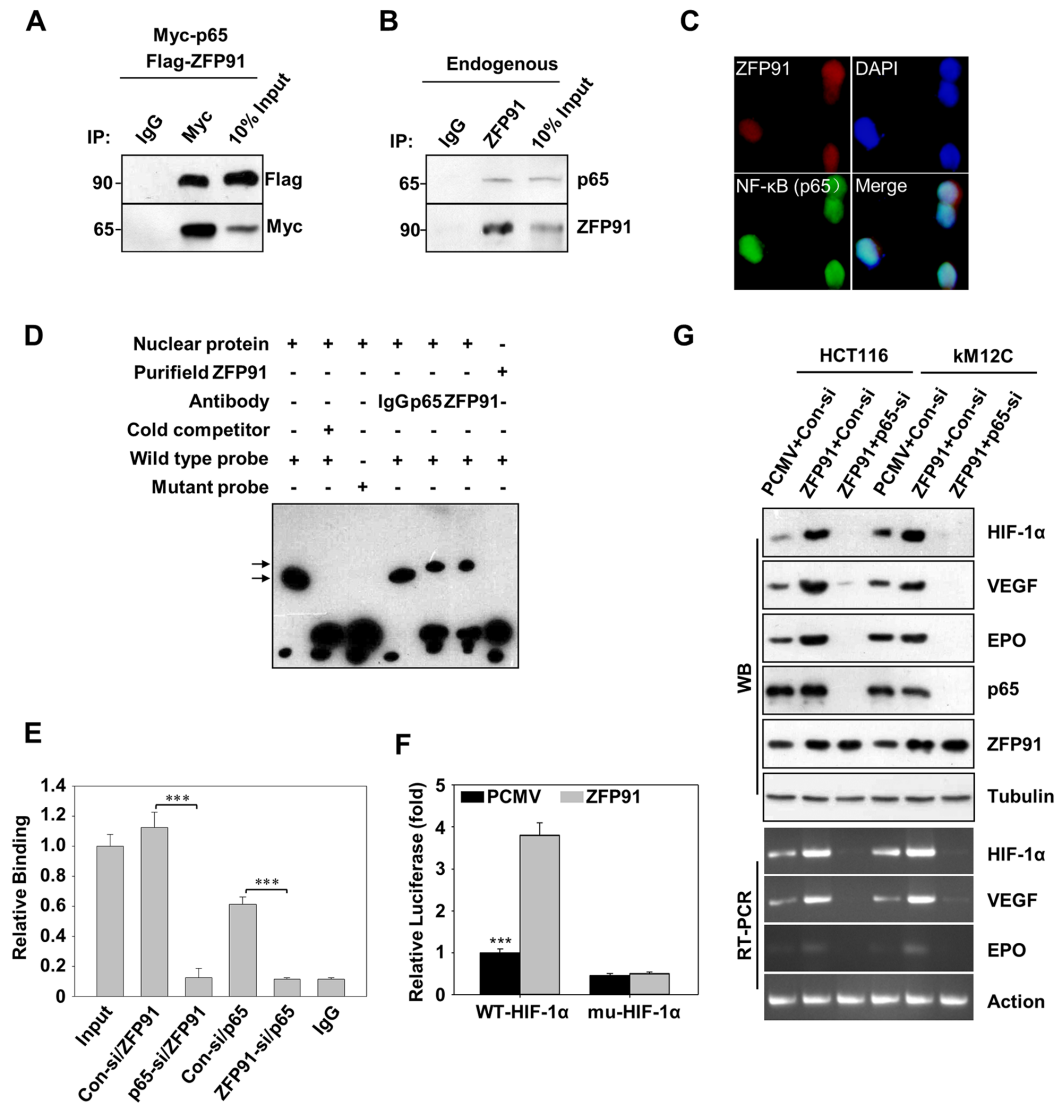
ZFP91 activates HIF-1α promoter through interacting with transcription factor NF-κB/p65 in colon cancer cells **A.** HCT116 cells co-transfected with Flag-ZFP91 and Myc-p65 stimulated with hypoxia for final 2 h. Cell lysates were immunoprecipitated with IgG and anti-Myc antibody, and the immune-complexes were analyzed using appropriate antibodies. **B.** The lysates of HCT116 cells stimulated with hypoxia for final 2 h were incubated with IgG and anti-ZFP91 antibody and the immune-complexes were analyzed using indicated antibodies. **C.** The nuclear localization of ZFP91 and NF-κB (p65) were detected by immunofluorescence staining in HCT116 cells under hypoxia for final 2 h. The images of ZFP91 (red) and NF-κB (p65) (green) were shown. DAPI staining (blue) was included to visualize the nucleus. **D.** HCT116 cells stimulated with hypoxia for final 2 h and prepared nuclear extracts for EMSA. The wild type HIF-1α promoter and mutant HIF-1α promoter were used as DNA probes. The shifts (upper arrow) were resulted from the addition of anti-ZFP91 and anti-p65 antibodies. IgG was used as a control antibody. **E.** HCT116 cells transfected with control siRNA, ZFP91 siRNA or p65 siRNA stimulated with hypoxia for final 2 h. ChIP analysis using antibodies against ZFP91, p65 or non-immune IgG and real-time PCR analysis using specific primers for the HIF-1α promoter and HIF-1α control region was performed. Data represented as mean ± standard deviation of three independent experiments (***p<0.001). **F.** HCT116 cells co-transfected with PCMV or Flag-ZFP91 together with PGL-WT-HIF-1α or PGL-mu-HIF-1α plasmids stimulated with hypoxia for final 2 h, and then promoter activity of HIF-1α was measured by luciferase reporter gene assay. Data represented as mean ± standard deviation of three independent experiments (^***^p<0.001). **G.** HCT116 and KM12C cells pre-treated with PCMV or Flag-ZFP91 together with control siRNA or NF-κB (p65) siRNA stimulated with hypoxia for final 2 h. The cell lysates were analyzed using indicated antibodies. The mRNA expression levels of HIF-1α VEGF and EPO were determined by RT-PCR.

Next, we tried to elucidate the underlying mechanism by which ZFP91 upregulates HIF-1α. Co-immunoprecipitation assays revealed that ZFP91 physically interacted with NF-κB/p65 upon co-expression of ZFP91 and NF-κB/p65 (Figure 3A), and endogenous interaction was observed under hypoxic condition (Figure 3B). This result was further confirmed by immunofluorescence assay indicating that ZFP91 and NF-κB/p65 were co-localized in the nucleus in HCT116 cells upon hypoxic stimulation (Figure 3C). NF-κB/p65 has been shown to interact with HIF-1α at an NF-κB consensus site in the HIF-1α promoter at −197/−188 base pairs from the initiation site [17, 20]. Therefore, we speculated that ZFP91 might bind to HIF-1α promoter at −197/−188 base pairs through interaction with NF-κB/p65 and involve in the transcriptional regulation of HIF-1α. Indeed, EMSA assays revealed that ZFP91 was able to bind to the HIF-1α promoter at −197/−188 base pairs. (Figure 3D, left first lane), whereas mutation of the NF-κB consensus site abrogated this response (Figure 3D, left third lane). Meanwhile, adding anti-p65 or anti-ZFP91 antibodies led to the supershift of interaction with the HIF-1α promoter DNA probe, suggesting that both NF-κB/p65 and ZFP91 bound to the HIF-1α promoter at −197/−188 base pairs (Figure 3D, right second and third lane). However, the purified recombinant ZFP91 alone did not bind to the HIF-1α promoter at −197/−188 base pairs by EMSA (Figure 3D, right first lane). ChIP assay revealed that the knockdown of NF-κB/p65 was able to dismiss the interaction of ZFP91 with HIF-1α promoter, suggesting that ZFP91 binds to HIF-1α promoter *via* interacting with NF-κB/p65. In addition, we also observed that the occupancy of NF-κB/p65 in HIF-1α promoter was disturbed when ZFP91 was silenced in the cells (Figure 3E), suggesting that ZFP91 might be an important coactivator in the NF-κB/p65-dependent HIF-1α transcription. Luciferase reporter gene assays revealed that ZFP91 failed to increase the activity of HIF-1α promoter when the NF-κB/p65 binding site was mutated (Figure 3F), further supporting that the biological function of ZFP91 on HIF-1α promoter is mediated *via* NF-κB/p65. Meanwhile, the knockdown of NF-κB/p65 by siRNA led to significant decrease of HIF-1α promoter activity and expression levels of HIF-1α, VEGF and EPO in HCT116 and KM12C cells (Supplementary Figure S1). Moreover, silencing of NF-κB/p65 was able to block the upregulations of HIF-1α, VEGF and EPO protein and mRNA levels mediated by ZFP91 in HCT116 and KM12C cells (Figure 3G). Thus, we concluded that ZFP91 is able to activate HIF-1α transcription through interacting with NF-κB/p65 in colon cancer cells.

### ZFP91 enhances the proliferation of colon cancer cells through HIF-1α in culture

**Figure 4:**
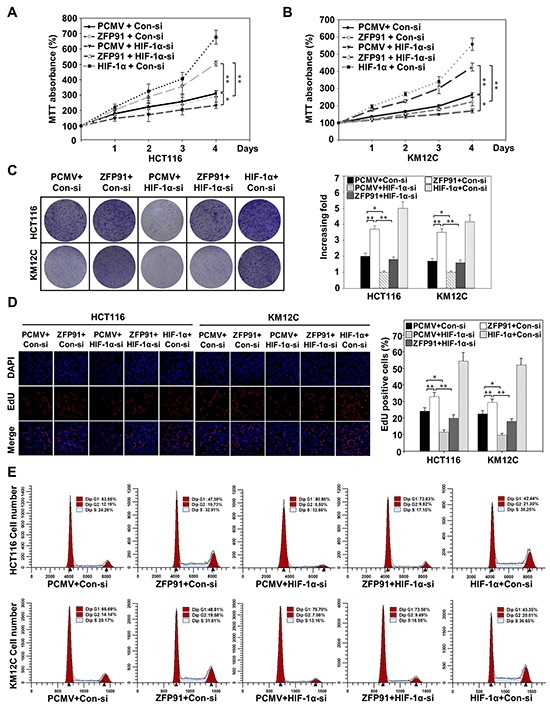
ZFP91 enhances the proliferation of colon cancer cells through HIF-1α in culture **A, B.** HCT116 (A) and KM12C (B) cells pre-treated with PCMV, Flag-ZFP91 or HA-HIF-1α together with control siRNA or HIF-1α siRNA, and subjected to MTT assay on days 1, 2, 3 and 4 under normoxic conditions. Data represented as mean ± standard deviation of three independent experiments (^*^p<0.05, ^**^p<0.01). **C, D.** HCT116 and KM12C cells pre-treated with PCMV, Flag-ZFP91 or HA-HIF-1α together with control siRNA or HIF-1α siRNA under normoxic conditions, and then cells proliferation was measured by colony formation assay (C) and EdU assay (D). Data represented as mean ± standard deviation of three independent experiments (^*^p<0.05, ^**^p<0.01). **E.** Flow cytometry analysis was performed to detect cell cycle of HCT116 and KM12C cells pre-treated with PCMV, Flag-ZFP91 or HA-HIF-1α together with control siRNA or HIF-1α siRNA under normoxic conditions.

Next, we examined whether HIF-1α involved in the promotion of proliferation of colon cancer cells mediated by ZFP91. MTT assays showed that the cell viability was significantly increased in ZFP91-overexpressing HCT116 and KM12C cells, as compared with control cells (Figure 4A and 4B), but slightly less than HIF-1α-overexpressing cells. These results were further confirmed by colony formation assay (Figure 4C). Interestingly, silencing HIF-1α strongly reduced cell proliferation in the ZFP91-overexpressing HCT116 and KM12C cells (Figure 4A, 4B, and 4C), suggesting that HIF-1α is involved in the ZFP91-triggered increase in cell proliferation. Comparable results were obtained in the EdU incorporation assays. As shown in Figure 4D, overexpression of ZFP91 in HCT116 and KM12C cells significantly increased the percentage of EdU positive cells, whereas knockdown of HIF-1α abolished ZFP91-triggered increase in the percentage of EdU positive cells. We next examined the effect of ZFP91 on cell cycle because HIF-1α is involved in the regulation of cell cycle [24]. Flow cytometry analysis showed that the overexpression of ZFP91 resulted in a robust increase of S-phase HCT116 cells from 24.26% to 32.91% (or KM12C cells from 20.17% to 31.61%, Figure 4E). While, the knockdown of HIF-1α reduced S-phase cells in ZFP91-overexpressing HCT116 (from 32.91% to 17.15%) and KM12C cells (from 31.61% to 16.95%). Our data suggests that ZFP91 promotes the proliferation of colon cancer cells through HIF-1α.

### ZFP91 promotes the tumor growth through HIF-1α *in vivo*

**Figure 5:**
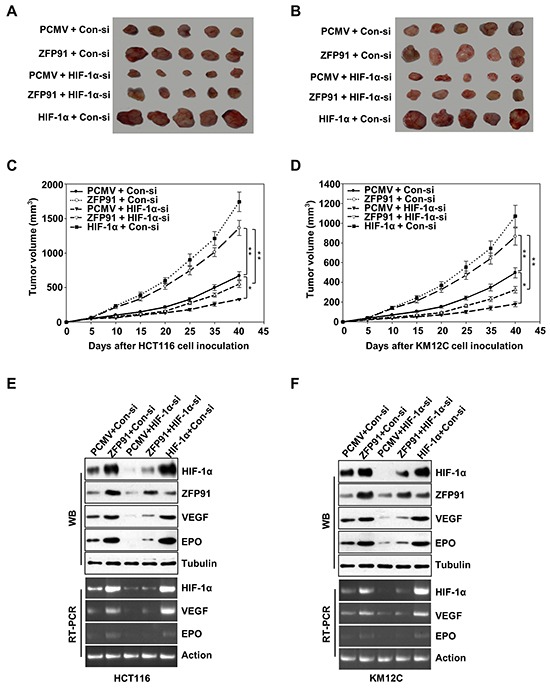
ZFP91 promotes the tumor growth through HIF-1α in mice **A, B.** HCT116 (A) and KM12C (B) cells pre-treated with PCMV, Flag-ZFP91 or HA-HIF-1α together with control siRNA or HIF-1α siRNA were implanted subcutaneously in the left flanks of nude mice. After 40 days, the tumors were dissected out from mice. **C, D.** The growth curve of tumor. Tumor size was measured every 5 days. Each point showed the mean ± SD (^*^p<0.05, ^**^p<0.01). **E, F.** The mean expression levels of HIF-1α, ZFP91, VEGF, and EPO in the tumor tissues from each group mice were determined by western blot and RT-PCR, respectively.

Based on our findings *in vitro*, we performed tumor xenograft assays and found that the overexpression of ZFP91 enhanced the growth of HCT116 and KM12C cells in nude mouse. Importantly, as the knockdown of HIF-1α dismissed the growth of colon cancer cells in mice, the knockdown of HIF-1α was able to block ZFP91-triggered growth rate increase of colon cancer cells in mice (Figure 5A–5D) and the mean expression levels of HIF-1α, VEGF and EPO protein and mRNA levels in each group of tumor tissue samples from mice (Figure 5E and 5F) were correlated well with the tumor volume of corresponding animal group. Furthermore, IHC assay showed that areas of strong ZFP91 signals displayed intense HIF-1α, VEGF and CD31, whereas areas with knockdown of HIF-1α exhibited lower HIF-1α, VEGF and CD31 staining signals in ZFP91-overexpressing colon cancer cells (Supplementary Figure S2). Taken together, we conclude that ZFP91 promotes the tumor growth through HIF-1α *in vivo*.

## DISCUSSION

This study advances our knowledge of the activation mechanism of HIF-1α and indicates that ZFP91 plays an important, yet previously unappreciated, role in the tumorigenesis and progression of colon cancer. The key findings of this study are that ZFP91 overexpression was significantly associated with progression of human colon cancer and that ZFP91 promotes colon cancer progression through upregulating HIF-1α in cooperation with NF-κB/p65. Given its function in non-canonical NF-κB pathway through NIK activation, ZFP91 could be a dual-functional oncoprotein in tumorigenesis.

Tumorigenesis is a complex multistep process, characterized by uncontrolled cell growth and tumor formation and is largely associated with progressive accumulation of genetic and various epigenetic alterations in genes or proteins that regulate cell proliferation [25, 26]. Therefore, identification of the genes and their products that lead to tumorigenesis is critical for providing new diagnostic and prognostic methods and potential therapeutic targets. Previously, it has been reported that ZFP91 is an important oncoprotein that is associated with the proliferation and tumorigenesis of many human cancer cells [3–6, 27]. Importantly, ZFP91 stabilizes and activates NIK, a crucial kinase for the non-canonical NF-κB pathway, which is associated with cancer growth and survival. Elevated expression of NIK and its role in oncogenic properties has been demonstrated in the proliferation and tumorigenesis of various solid tumors including lung cancer, pancreatic cancer, ovarian cancer and colon cancer [28–30]. With respect to previous reports, it is reasonable to suggest that ZFP91 could be associated with cancer at least through NIK activation, however, our study reveals previously unappreciated clinical significance and biological role of ZFP91 in cancer. We found that ZFP91 was significantly upregulated in a large cohort of human colon cancer tissues.

Mounting evidences have established that HIF-1α plays an important role in tumorigenesis *via* the regulation of a variety of biological processes, such as proliferation, angiogenesis, invasion, metastasis, and cell cycle progression [24, 31, 32]. Indeed, upregulation of HIF-1α expression is observed in multiple cancers, including colon cancer, breast cancer, prostate cancer, chronic myelogenous leukemia, glioblastoma, rhabdomyosarcoma, and leukemia [33–35]. In this regard, we found another crucial role of ZFP91 other than NIK activation in cancer: ZFP91 was able to dramatically upregulate the expression levels of HIF-1α in colon cancer cells. Consequently, ZFP91 elevated the expression of VEGF and EPO. These results drove us to determine the mechanisms by which ZFP91 upregulates HIF-1α expression. Notably, we observed the nucleus localization of ZFP91 in colon cancer cells, implying that ZFP91 may exert nuclear functions. ZFP91 contains the functional domain of leucine-zipper pattern, implying its involvements in gene transcription regulations [3]. Accordingly, we investigated whether ZFP91 is able to interact with specific transcriptional factors involved in the transcription of HIF-1α gene. We indeed observed that ZFP91 is recruited to the HIF-1α promoter, which contains a NF-κB site at −197/−188 base pairs, of which previously demonstrated as the binding site of NF-κB/p65 [15, 19, 20]. Further investigations with ChIP assay identified that NF-κB/p65 was involved in the interaction between ZFP91 and HIF-1α promoter. Many studies characterized the interactions of NF-κB/p65 with several nuclear cofactors including p53, Sp1, P300, HDACs, myc, Stat3, Hsp90 and other proteins in multiple physiological and pathological processes including inflammatory and immune response, cellular stress reactions, carcinogenesis, cell survival and apoptosis [36, 37]. We found that ZFP91 interacts with the NF-κB/p65 that is required not only for the binding of ZFP91 to the HIF-1α promoter but for the transcriptional activation of HIF-1α gene mediated by ZFP91. More importantly, our finding suggests that ZFP91 may serve as a transactivator to play crucial roles in the development of colon cancer.

In summary, we report that ZFP91 promotes colon cancer progression through upregulating HIF-1α *via* NF-κB/p65. Our results suggest that ZFP91 may serve as a driver gene in the development of cancer and has potential as a relevant clinical indicator of disease progression and as a prognostic marker for patient survival in human colon cancer. More understanding of the precise role of ZFP91 in human cancer may provide the opportunity to develop a novel therapeutic strategy by suppressing expression of ZFP91 in colon cancer cells.

## MATERIALS AND METHODS

### Cell culture, transfection and luciferase reporter assay

Human colon cancer cell lines HCT116 and KM12C were grown in RPMI or DMEM with penicillin (100 units/ml)-streptomycin (100 units/ml) (Invitrogen) and 10% heat-inactivated fetal bovine serum (Hyclone). Transfections were performed using Lipofectamine 2000 (Invitrogen). HIF-1α promoter luciferase activity was measured using the Dual Luciferase Reporter Assay system.

### Plasmids and retroviral infection

The complete coding region of human ZFP91, p65 and HIF-1α cDNA was amplified from human gastric cancer cell line SNU-638 cDNA library by PCR. We generated Flag-ZFP91, Myc-p65 and HA-HIF-1α by PCR and cloned into pCMV-Tag2B (Stratagene), pCMV-Tag1 (Stratagene) and pCMV-HA (Clontech), respectively. Human ZFP91-specific siRNA (5′-CCAGGTGGCATTAGTAGTGAA-3′), HIF-1α-specific siRNA (5′-GTGGTTGGATCTAACACTA-3′) and a scrambled control siRNA (5′-AAGGAGACGAG CAAGAGAA-3′) oligonucleotides cloned into pSuper-retro-puro vector. Retroviral production and infection were conducted as previously described [38]. Human p65-specific siRNA sequences were (5′-GCTGATGTGCACCGACAAG-3′). The upstream region (from −538 to +284) of HIF-1α gene was amplified by PCR from HCT116 cells, inserted into the KpnI/HindIII site in the pGL3-basic vector and named pGL3-WT-HIF-1α. Mutant construction of-197/−188 region of HIF-1α promoter named pGL3-mu-HIF-1α, carried a substitution of four nucleotides within the binding site of NF-κB/p65. Mutagenesis primers used were as follows: 5′-gggctggggtATTAacttgccgcctgcgtcg ctcgc-3′and 5′-gcgagcgacgcaggcggcaagtTAATaccccagccc-3′.

### Patient information and tissue specimens

This study was conducted on a total of 88 paraffin-embedded, archived colon cancer samples, which were histopathologically at the Cancer Center, Yanhua Hospital (Beijing, China). For the use of these clinical materials for research purposes, prior consent of the patients and approval from the Institutional Research Ethics Committee were obtained.

### Immunohistochemical analysis

Sections (4-5 μm) of paraffin-embedded colon cancer specimens were prepared and standard immunohistochemical procedures were carried out using a polyclonal anti-ZFP91 antibody as previously described [5] (1:50 dilution) and a monoclonal anti-HIF-1α antibody (BD, Biosciences; 1:25 dilution).

### Immunoprecipitation and immunoblotting

Cells lysates in lysis buffer (50 mM Tris, pH 7.4, 150 mM NaCl, 1 mM EDTA, 1% Triton X-100 and protease inhibitors cocktail) were centrifuged at 15,000 rpm for 30 minutes at 4°C, and 1 mg protein of cleared lysates were used for each immunoprecipitation. The lysates were incubated overnight at 4°C with primary antibodies and then 30 μl of protein A/G PLUS-agarose beads (Santa Cruz) were added and incubation continued. Beads were washed with cold lysis buffer and boiled in SDS-PAGE sample buffer before elctrophoresis. Primary antibodies used were anti-ZFP91 [5], anti-HIF-1α (BD Biosciences; 610959 and Novus Biologicals; NB100-105), anti-VEGF (Santa Cruz; sc-152), anti-EPO (Santa Cruz; sc-80995), anti-CD31 (Santa Cruz; sc-1506), anti-p65 (Santa Cruz; sc-109), anti-Topo-I (Santa Cruz; sc-5342), anti-α-tubulin (Sigma; T5168).

### RT-PCR analysis

Total RNA from HCT116 and KM12C were obtained using RNA Mini kit (Qiagen, Valencia, CA, USA). Total RNA (2 μg) was used to perform reverse transcription-PCR (RT-PCR) using RT-PCR kit (Invitrogen, Carlsbad, California, USA) according to the manufacturer's protocol. The PCR primers for VEGF were 5′-GCTCTACCTCCACC ATGCCAA-3′ (sense) and 5′-TGGAAGATGTCCACCAG

GGTC-3′ (antisense); for EPO were 5′-CACTT TCCGCAAACTCTTCCG-3′(sense) and 5′-GTCAC AGCTTGCCACCTAAG-3′ (antisense); for HIF-1α were 5′-CTCAAAGTCCGACAGCCTCA-3′ (sense) and 5′-CCCTGCAGTAGGTTTCTG

CT-3′ (antisense); for ZFP91 were 5′-AGCTACCA TTTGCCTACAA-3′ (sense) and 5′-GGGAAACG GCTGAGATAGTTT-3′ (antisense); for GAPDH were 5′-ACCACA

GTCCATGCCATCAC-3′ (sense) and 5′-TCCAC CACCCTGTTGCTGTA-3′ (antisense). The oligonucleotide sequences of the reaction products were confirmed by sequencing.

### Cell growth and viability assays

Cells were seeded in 96-well plates (1×10^4^ cells/ml) overnight for MTT assay or 6-well plates (5×10^3^ cells/plate) for 10 days for colony formation assay. The colonies were stained with 1% crystal violet for 30 seconds after fixation with 10% formaldehyde for 5 minutes.

### EdU labeling and immunofluorescence

Cells were plated on coverslips (Fisher Scientific; 12-545-80). After 24 hours, cells were incubated with 5-ethynyl-2′-deoxyuridine (EdU, RIBOBIO; R11053) for 1 hour and stained with Apollo®567 according to the manufacturer's instruction. The stained cells were observed with Olympus IX83 inverted fluorescence microscope.

### Electrophoretic mobility shift assay (EMSA)

EMSA for HIF-1α promoter was performed using the LightshiftChemiluminescentEMSA kit (Pierce; 20148). Briefly, DNA was biotin labeled using the biotin 3′-end-labeling kit (Pierce; 89818) in a 50 μl reaction buffer and 5 pmol of double-stranded HIF-1α promoter oligonucleotide (5′-ggggtggggacttgccgcctgcg-3′ and 5′-cgcaggcggcaagtccccacccc-3′) or 5 pmol of double-stranded HIF-1α mutant promoter oligonucleotide (5′-ggggtattaacttgccgcctgcg-3′ and 5′-cgcaggcggcaagttaatacccc-3′) incubated with 10μl of 5×terminal deoxynucleotidyltransferase buffer, 5 μl of 5 μM biotin-N4-CTP, 10 units of diluted terminal deoxynucleotidyltransferase, and 25 μl of ultrapure water at 37°C for 30 minutes. Each binding reaction contained 1×binding buffer [100 mM Tris, 500 mM KCl, and 10 mM DTT (pH 7.5)], 2.5% glycerol, 5mM MgCl_2_, 50 ng/μl poly(deoxyinosinic-deoxycytidylic acid), 0.05% NP40, 2.5 μg of nuclear extract, and 20 femtomoles of biotin-end-labeled target DNA. The contents were incubated for 20 minutes, added 5 μl of 5× loading buffer, subjected to gel electrophoresis. After transfer to a nylon membrane, DNA was cross-linked to the membrane using a UV cross-linker. The biotin-end-labeled DNA was detected using streptavidin-horseradish peroxidase conjugate and a chemiluminescent substrate. The membrane was exposed to X-ray film. For supershift assay, antibodies against p65 or ZFP91 were added and incubation was continued for an additional 30 minutes.

### Chromatin immunoprecipitation

Chromatin immunoprecipitation (ChIP) assay was performed using ChIP kit from Millipore (Millipore; 17-295). HCT116 cells were transfected with control siRNA, ZFP91 siRNA or p65 siRNA stimulated with hypoxia for final 2 h, and fixed by adding formaldehyde to the medium. After cross-linking, glycine was added at a final concentration of 125 mM, and the cells were harvested with lysis buffer. The cell nuclei sub-fractions were pelleted by centrifugation and resuspended in nuclear lysis buffer. The nuclear lysates were sonicated to generate DNA fragments of 0.5-1 kb, and then ChIP were performed with antibodies against ZFP91 or p65. Real-time PCR amplification was performed with DNA extracted from the ChIP assay and primers flanking the NF-κB binding site in promoter region of HIF-1α gene. The primers used in ChIP analysis were as follows: HIF-1α gene's promoter were 5′-GAACAGAGAGCCCAGCAGAG-3′ and 5′-TGTGCACTGAGGAGCTGAGG-3′; The HIF-1α control were 5′-TGCTCATCAGTTGCCACTTC-3′ and 5′-AAAACATTGCGACCACCTTC-3′.

### Flow cytometry analysis

Cells were harvested, washed with cold phosphate-buffered saline, and processed for cell-cycle analysis by flow cytometry. Briefly, the cells were fixed in 75% ethanol, stained with RNase A (20μg/ml) and propidium iodide (50 μg/ml) in phosphate-buffered saline, incubated for 30 minutes at 37°C in the dark and analyzed by a BD Accuri™ C6 instrument (BD, Biosciences) with Modifit LT 4.0 trial cell cycle analysis software.

### Xenografted tumor model

All experimental procedures were approved by the Institutional Animal Care and Use Committee of Yanbian University. The female Balb/c nude mice (4-5 weeks of age, 18-20 g, Vital River Laboratory Animal BeiJing, China) were randomly divided into 10 groups (n=8/group). For tumor cell implantation, HCT116 and KM12C cells pre-treated with PCMV, Flag-ZFP91 or HA-HIF-1α together with control siRNA or HIF-1α siRNA were harvested and 1×10^7^ cells in 200 μl phosphate-buffered saline were subcutaneously injected into the mice. Tumor size was measured in two dimension with calipers every 5 days, up to 40 days after injection. Tumor volume was calculated using the equation: (length×(width)^2^)/2.

### Statistical analysis

All statistical analyses were conducted by using the SPSS version 13.0 statistical software packages. Comparisons between groups for statistical significance were carried out with a 2-tailed paired Student *t* test. The relationship between ZFP91 or HIF-1α expression was analyzed by the χ^2^ test. A *p* value of less than 0.05 was considered statistically significant in all cases.

## SUPPLEMENTARY FIGURES AND TABLES


