# Supplementary material for: Zinc finger protein 91 (ZFP91) activates HIF-1α via NF-κB/p65 to promote proliferation and tumorigenesis of colon cancer
Source: Oncotarget. 2016 Apr 28;7(24):36551–62. doi: 10.18632/oncotarget.9070 (PMC5095020; doi:10.18632/oncotarget.9070)
Supplement: Supplementary file 1 [file oncotarget-07-36551-s001.pdf]

SUPPLEMENTARY FIGURES AND TABLES

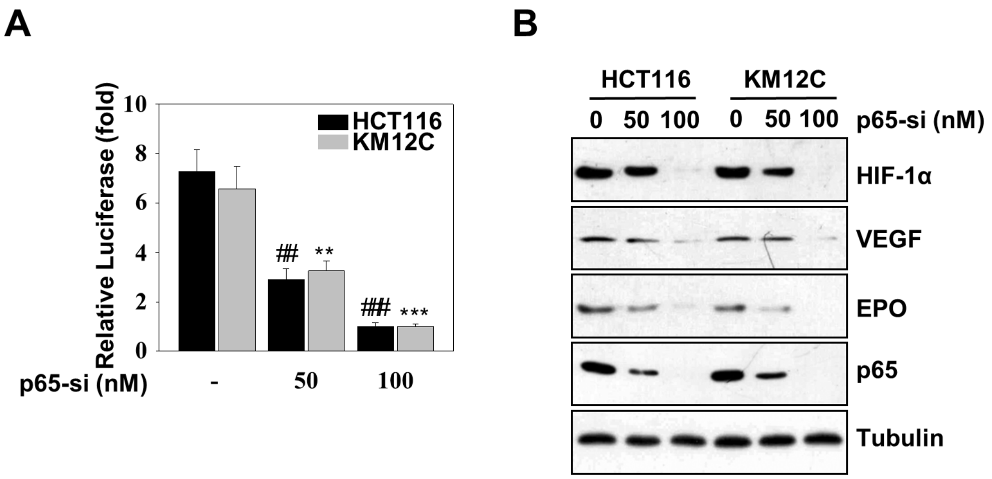

Supplementary Figure S1: A. the promoter activity of HIF-1α was examined by luciferase reporter gene assay in HCT116 and KM12C cells when the cells were treated with p65 siRNA (50 or 100 nM) (**p<0.01, ***p<0.001, ##p<0.01, ###p<0.001). **B.** HCT116 and KM12C cells were treated with p65 siRNA (50 or 100 nM). The cell lysates were analyzed by immunoblot using indicated antibodies for HIF-1α, VEGF, EPO, p65 and Topo-I.

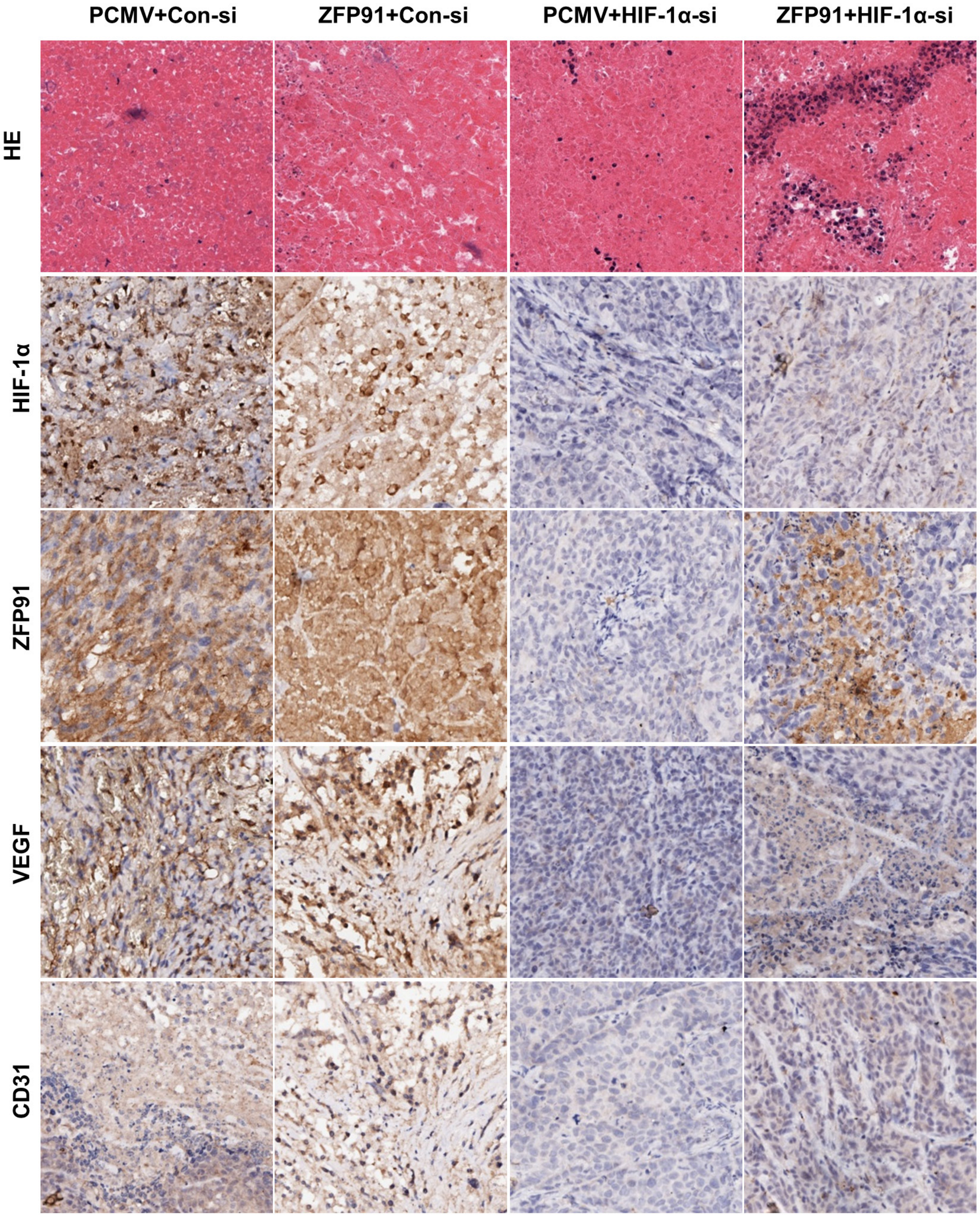

Supplementary Figure S2: H&E or IHC analysis of ZFP91, HIF-1α, VEGF and CD31 expression in tumor xenograft tissues. Original magnification, 200x.

Supplementary Table S1: Correlation analysis of ZFP91 expression and clinicopathological features in colon cancer

**Variable**	**ZFP91 expression**			
	**All cases**	**Negative**	**Positive**	***p* value***
Age, y				0.326
≤65	34	8 (23.5%)	26 (76.5%)	
>65	54	18 (33.3%)	36 (66.7%)	
Sex				0.426
Male	45	15 (33.3%)	30 (66.7%)	
Female	43	11 (25.6%)	32 (74.4%)	
Tumor size, cm				0.848
≤5	46	14 (30.4%)	32 (69.6%)	
>5	42	12 (28.6%)	30 (71.4%)	
Clinical stage				0.020
I	7	1 (14.3%)	6 (85.7%)	
II	37	17 (45.9%)	20 (54.1%)	
III	36	8 (22.2%)	28 (77.8%)	
IV	8	0 (0%)	8 (100%)	
Differentiation				0.386
Well	8	1 (12.5%)	7 (87.5%)	
Moderate	50	14 (28%)	36 (72%)	
Poor	30	11 (36.7%)	19 (63.3%)	
T classification				0.019
T1	14	1 (7.1%)	13 (92.9%)	
T2	17	2 (11.8%)	15 (88.2%)	
T3	44	19 (43.2%)	25 (56.8%)	
T4	13	4 (30.7%)	9 (69.3%)	
N classification				0.049
N0	40	17 (42.5%)	23 (57.5%)	
N1	29	6 (20.7%)	23 (79.3%)	
N2	19	3 (15.8%)	16 (84.2%)	
M classification				0.055
M0	80	26 (32.5%)	54 (67.5%)	
M1	8	0 (0%)	8 (100%)	

*x^2 test

Supplementary Table S2: Correlation analysis of HIF-1α expression and clinicopathological features in colon cancer

Variable	HIF-1α expression			
	**All cases**	**Negative**	**Positive**	***p* value***
Age, y				0.921
≤65	34	18 (52.9%)	16 (47.1%)	
>65	54	28 (51.9%)	26 (48.1%)	
Sex				0.290
Male	45	26 (57.8%)	19 (42.2%)	
Female	43	20 (46.5%)	23 (53.5%)	
Tumor size, cm				0.091
≤5	46	28 (60.9%)	18 (39.1%)	
>5	42	18 (42.9%)	24 (57.1%)	
Clinical stage				0.005
I	7	4 (57.1%)	3 (42.9%)	
II	37	25 (67.6%)	12 (32.4%)	
III	36	17 (47.2%)	19 (52.8%)	
IV	8	0 (0%)	8 (100%)	
Differentiation				0.246
Well	8	5 (62.5%)	3 (37.5%)	
Moderate	50	29 (58%)	21 (42%)	
Poor	30	12 (40%)	18 (60%)	
T classification				0.024
T1	14	3 (21.4%)	11 (78.6%)	
T2	17	7 (41.2%)	10 (58.8%)	
T3	44	29 (65.9%)	15 (34.1%)	
T4	13	7 (53.8%)	6 (46.2%)	
N classification				0.049
N0	40	26 (65%)	14 (35%)	
N1	29	14 (48.3%)	15 (51.7%)	
N2	19	6 (31.6%)	13 (68.4%)	
M classification				0.002
M0	80	46 (57.5%)	34 (42.5%)	
M1	8	0 (0%)	8 (100%)	
*x^2 test				
